# Personalia

**Published:** 1914-03-14

**Authors:** 


### Personalia.

Mr. J. S. Horsley has been elected hon. secretary of the Brighton, Hove, and Preston Provident Dispensary, in place of the Rev. E. H. Enys, who has resigned.

Lieut.-Col. R. P. Simpson has resigned the hon. secretaryship of Petworth Cottage Hospital, which he has held for nineteen years. Dr. Eardley Wilmot has been elected to succeed him.

The Mental Hospitals Committee of the London County Council have recently granted permission to Dr. Bonnichsen, a doctor of medicine of Copenhagen, to work as a resident clinical assistant at Hanwell Mental Hospital for a period of two months without salary.

Dr. James Hill, M.B., Ch.B., B.A.O. (Belfast), has been promoted by the Camberwell Board of Guardians to the position of assistant medical officer at the Children's Homes and the Infirmary, which was vacant by the resignation of Dr. C. H. L. Rixon. Dr. Hill, who was house surgeon and ophthalmic officer at the Royal Infirmary, Sheffield, in 1913, previously held the post of assistant medical officer at Gordon Road Workhouse, Camberwell.

